# Intramedullary sclerosing meningioma: operative video

**DOI:** 10.3171/2023.7.FOCVID2385

**Published:** 2023-10-01

**Authors:** Giada Garufi, Giuseppe Ricciardo, Alfredo Conti, Salvatore Massimiliano Cardali

**Affiliations:** 1 Department of Neurosurgery, Azienda Ospedaliera Papardo, University of Messina, Italy;; 2 Department of Neurosurgery, Mayo Clinic, Rochester, Minnesota; and; 3 Dipartimento di Scienze Biomediche e Neuromotorie (DIBINEM), Alma Mater Studiorum Università di Bologna, Italy

**Keywords:** fluorescein, sclerosing, meningioma

## Abstract

Sclerosing meningiomas (SMs) represent a rare histological variant of meningiomas, first described in 1989 as invasive bulking masses of whorling collagen bundles with a minimum percentage of meningothelia-resembling cells, and they are often misdiagnosed. The literature reports only 30 cases of SMs, with only two of them being intramedullary. The authors present the case of a patient with a cervical intramedullary SM who presented with gait disturbances, sensory deficits, weakness in four extremities, and hyperreflexia. The surgery was performed under neurophysiological monitoring and after administration of sodium fluorescein, which allowed us to discriminate the exact myelotomy point. Intramedullary SMs are very rare entities whose correct management may result in a good outcome.

## Transcript

We present here a case of a 66-year-old woman who was admitted to our institution with a 6-month history of progressive extremities weakness, paresthesias of both superior arms, worse on the right side, followed by gait and balance disorders. She has a history of meningiomatosis where a huge convexity meningioma was operated 5 years early without any neurological impairment. At the neurological examination, the patient exhibited a decreased general muscle strength with a 3/5 MRC scale quadriparesis, a bilateral C4–5 territory hypesthesia. Babinski reflex was positive with a generalized hyperreflexia of deep tendon reflexes. Romberg test was positive.

### 1:44 Neuroradiological Findings.

The total spine MRI showed an intradural intramedullary lesion at C4–5 level. The lesion was hyperintense in T1- and hypointense in T2-weighted sequences. After administration of gadolinium, the lesion demonstrated heterogeneous contrast enhancement with a hypointense core. The benign nature of the vast majority of meningiomas defines the surgical strategies as the gold standard treatment of these lesions. Gross-total resection with a one-step surgery is curative.^1,2^ In case of high risk of surgical morbidity, less invasive treatments are justified and they are tailored on the specific lesion in order to achieve the best surgical outcome and the best prognosis for the patient. Modern functional neurosurgical tools as intraoperative neurophysiological monitoring could give the surgeon the opportunity to reduce neurological deficit guaranteeing the gross-total resection. The use of fluoroscopes during the procedure could improve the curative goal of surgical approach. Benefits of the surgical procedure are biopsy and diagnosis, gross-total resection with no need of other treatments, neurological function recovery, arrest of further progression. Risks are vascular injuries, neurological impairment to achieve radical surgery, CSF leak, wound complications. Alternatives are the neuroradiological follow-up during time. The surgery was made with a patient in a prone position with a preoperative administration of fluorescein. Preoperative x-ray could help us to localize the lesion level. The surgery was made with the intraoperative neurophysiological monitoring. The patient underwent a posterior bilateral C4–5 hemilaminectomy and microscopic exeresis of the cervical lesion.

### 4:14 Administration of Fluorescein.

The exeresis was performed under neurophysiological intraoperative D-wave, MEPs and SEPs monitoring, and after administration of sodium fluorescein, which allows us to intraoperatively discriminate the intra-axial nature of the lesion. Tumor was found to be white-grayish, pretty avascular, with a hard consistency and without an arachnoidal plane among the medullary parenchyma, especially in the ventral plane. The intraoperative pathological examination documented a paucicellular sclerotic bulk mass with a moderate vascular component.

### 7:08 Exeresis of the Lesion.

The microscopic analysis demonstrated a hypocellular proliferation with a sclerotic stroma, a scanty population intermingled by cellular areas with spindle elements, arranged in intersecting fascicles and a variable collagen stroma. The immunohistochemical evaluation demonstrated a strong stain for vimentin, focal positivity for epithelial membrane antigen, positive nuclear staining for progesterone receptors, and a very low proliferation demonstrated by Ki-67 < 2%.^3,4^ Intramedullary sclerosing meningiomas are not mentioned in the WHO 2021 CNS tumor classification, and they are included into other morphological variation subtype group. As far as WHO 2016 added the criterion of brain invasion to atypical meningioma diagnosis, we must reconsider their clinical significance.^3^

### 8:11 Outcome: MRI and Clinical Evaluation.

The postoperative MRI demonstrated the residual surgical cave after the administration of gadolinium.^5^ The 6-month postoperative neurological examination showed an improved four-extremities strength with no alteration in the segmental proofs. Sensory alterations were improved with no gait and balance disorders complaint. Romberg test was negative. Paresthesias were not described by the patient.

## Disclosures

The authors report no conflict of interest concerning the materials or methods used in this study or the findings specified in this publication.

## Author Contributions

Primary surgeon: Cardali. Assistant surgeon: Garufi, Ricciardo. Editing and drafting the video and abstract: Garufi, Ricciardo, Conti. Critically revising the work: Garufi, Conti. Reviewed submitted version of the work: Garufi. Approved the final version of the work on behalf of all authors: Garufi. Supervision: Garufi.

## Supplemental Information

### Patient Informed Consent

The necessary patient informed consent was obtained in this study.
